# BAG1 down‐regulation increases chemo‐sensitivity of acute lymphoblastic leukaemia cells

**DOI:** 10.1111/jcmm.16822

**Published:** 2021-08-17

**Authors:** Elena Mariotto, Diana Corallo, Marcella Pantile, Emanuela Giarin, Martina Pigazzi, Giuseppe Basso, Giampietro Viola, Sanja Aveic

**Affiliations:** ^1^ Department of Woman's and Child's Health, Haematology‐Oncology Clinic and Lab University of Padova Padova Italy; ^2^ Neuroblastoma Laboratory Fondazione Istituto di Ricerca Pediatrica Città della Speranza Padova Italy; ^3^ Pediatric hematology, oncology and hematopoietic cell&gene therapy Fondazione Istituto di Ricerca Pediatrica ‐ Città della Speranza Padova Italy; ^4^ Department of Dental Materials and Biomaterials Research RWTH Aachen University Hospital Aachen Germany

**Keywords:** BAG1, B‐ALL, BCL2, leukaemia

## Abstract

BCL2‐associated athanogene‐1 (BAG1) is a multi‐functional protein that is found deregulated in several solid cancers and in paediatric acute myeloid leukaemia. The investigation of BAG1 isoforms expression and intracellular localization in B‐cell acute lymphoblastic leukaemia (B‐ALL) patient‐derived specimens revealed that BAG1 levels decrease during disease remission, compared to diagnosis, but drastically increase at relapse. In particular, at diagnosis both BAG1‐L and BAG1‐M isoforms are mainly nuclear, while during remission the localization pattern changes, having BAG1‐M almost exclusively in the cytosol indicating its potential cytoprotective role in B‐ALL. In addition, knockdown of BAG1/BAG3 induces cell apoptosis and G1‐phase cell cycle arrest and, more intriguingly, shapes cell response to chemotherapy. BAG1‐depleted cells show an increased sensitivity to the common chemotherapeutic agents, dexamethasone or daunorubicin, and to the BCL2 inhibitor ABT‐737. Moreover, the BAG1 inhibitor Thio‐2 induces a cytotoxic effect on RS4;11 cells both in vitro and in a zebrafish xenograft model and strongly synergizes with pan‐BCL inhibitors. Collectively, these data sustain BAG1 deregulation as a critical event in assuring survival advantage to B‐ALL cells.

## 1 INTRODUCTION

B‐cell acute lymphoblastic leukaemia (B‐ALL) is the most common childhood malignancy. As described for many cancer types, leukaemic cells acquire disruptions of normal signal transduction while evolving strategies to circumvent programmed cell death induction ^1^, ^2^. Among the most studied apoptosis‐related proteins are BCL2 family members. The dominance of the anti‐apoptotic proteins of the family (such as BCL2, MCL1 and BCL_XL_) is frequently encountered in malignant cells ^3^.

The members of the BCL2‐associated athanogene (BAG) protein family sustain the anti‐apoptotic function of BCL2. Their altered expression was reported in either solid tumours or leukaemias ^4^. A human BAG1 protein has three major isoforms: nuclear BAG1‐L (50 kDa), nuclear/cytosolic BAG1‐M (46 kDa) and predominantly cytosolic BAG1‐S (36 kDa) ^5^, ^6^. In the last two decades, several cancer‐related studies attributed a pro‐malignant connotation to either over‐expression and deregulation of BAG1 protein or the variation in the intracellular localization of BAG1 isoforms ^7^. From these studies, the role of BAG1 in protecting the cells from pro‐apoptotic stimuli induced by drug or radiation therapies is emerging ^8^. The same function was described for paediatric acute myeloid leukaemia (AML), where BAG1 over‐expression sustained leukaemic phenotype ^9^. However, in these cells, a compensatory mechanism between BAG1 and BAG3 was reported ^10^. Hence, a concomitant down‐regulation of BAG1 and BAG3 was necessary for triggering AML cell death. The involvement of BAG1 in paediatric B‐ALL is poorly defined, whereas other pro‐apoptotic BCL2 family proteins have been examined extensively as molecular therapeutic targets. At the same time, BAG3 protein has been confirmed as highly expressed in primary ALL specimens and cell lines, regulating their survival ^11^.

## 2 RESULTS AND DISCUSSION

**FIGURE 1:**
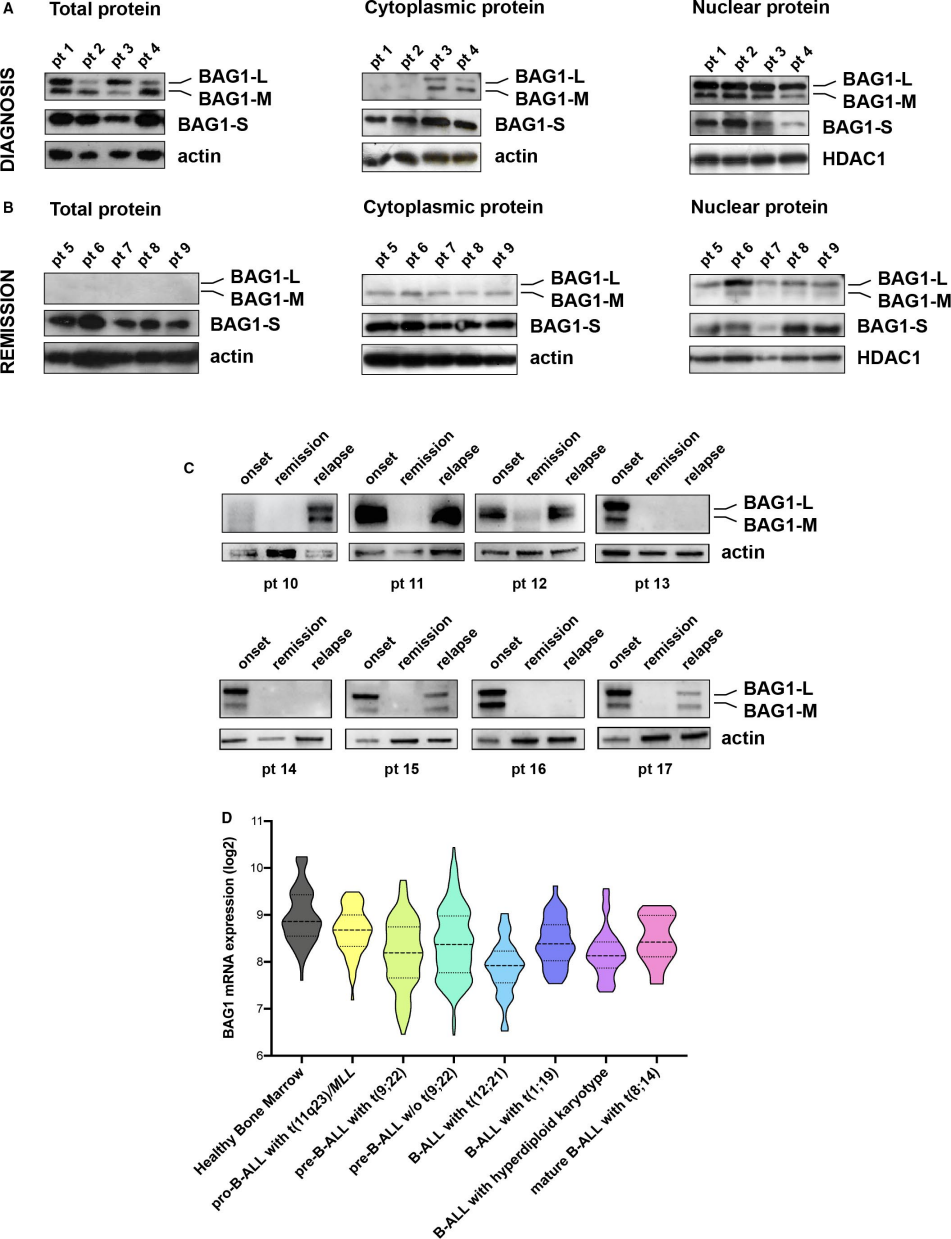
BAG1 expression levels. Western blot analysis of BAG1 protein expression levels in paediatric B‐ALL patients (pt) at first diagnosis (A) and remission stage (B) in the total protein lysate, cytoplasmic and nuclear fraction. While the total protein shows that the longest BAG1 isoforms (BAG1‐M and BAG1‐L) are highly expressed at diagnosis and predominantly localized in the nuclear fraction, their expression is almost undetectable at remission in the total protein fraction. The smaller BAG1‐S isoform expression is consistent in both stages. (C) Representative Western blot analysis of BAG1‐M and BAG1‐L isoform in paired B‐ALL specimens collected at disease onset, remission and relapse. Majority of analysed specimens show high BAG1‐L level at diagnosis and during relapse. Expression of BAG1‐S is included in these results in Figure S1. (D) Gene expression data for BAG1 mRNA in B‐ALL paediatric patients (*n* = 558) and healthy bone marrow (*n* = 71) collected from the Interlaboratory Microarray Innovations in LEukemia (MILE) study (GSE13204, https://doi.org/10.1200/JCO.2009.23.4732). Y‐axis represents the BAG1 probe (211475_s_at) signal on log2 scale. The 211475_s_at probe maps a mRNA region common to all BAG1 isoforms. 1^st^ quartile, median and 3^rd^ quartile are depicted for each group. Welch ANOVA test with Dunnett's correction for multiple comparisons was performed for each B‐ALL subgroup compared to healthy bone marrow, and the multiplicity‐adjusted *p* values were pro‐B‐ALL with t(11q23)/*MLL* (*n* = 69, *p* = 0.045), pre‐B‐ALL with t(9;22) (*n* = 116, *p* < 0.0001), pre‐B‐ALL w/o t(9;22) (*n* = 231, *p* < 0.0001), B‐ALL with t(12;21) (*n* = 57, *p* < 0.0001), B‐ALL with t(1;19) (*n* = 36, *p* < 0.0001), B‐ALL with hyperdiploid karyotype (*n* = 38, *p* < 0.0001) and mature B‐ALL with t(8;14) (*n* = 11, *p* = 0.08). Abbreviations: B‐ALL, B‐cell acute lymphoblastic leukaemia; pt, patient

To better characterize the role of BAG1 in childhood ALL, we assessed BAG1’s protein profiles in the bone marrow (BM) aspirates collected at different stages of the disease (Table S1). As shown in Figure 1A, BAG1‐L and BAG1‐M isoforms are predominantly concentrated in the nuclear protein fraction. We detected a substantial reduction in BAG1 protein levels within the total protein fraction, particularly of BAG1‐L and BAG1‐M isoforms, in the samples collected during disease remission (Figure 1B), resembling previously reported expression profiles in healthy BM ^9^. Since the three BAG1 isoforms can be found within diverse sub‐cellular portions, the expression of BAG1 protein isoforms was investigated in cytosol and nucleus. At diagnosis, BAG1‐L and BAG1‐M were almost exclusively nuclear (Figure 1A) while during remission BAG1‐M was excluded from the nucleus (Figure 1B). These findings sustain that even in B‐ALL, like in other solid cancers, either over‐expression or shuttling of BAG1‐L and BAG1‐M isoforms within the cell might sustain disease progression ^12^, ^13^. These data were further corroborated by examining BAG1‐L and BAG1‐M isoforms in matched B‐ALL specimens collected at diagnosis, remission and relapse. A significant reduction in BAG1‐L and BAG1‐M isoforms at the remission stage was followed by a marked increase at relapse (Figure 1C and Figure S1). Hence, it is plausible that either protein abundance or the localization of specific BAG1 isoforms may determine drug resistance in B‐ALL cells sustaining tumour recurrence. This might justify why BAG1 has been neglected so far by comprehensive B‐ALL transcriptomic analysis using probes mapping the BAG1 transcript, which is common to all BAG1 isoforms (Figure 1D). Collectively, these data further highlight the importance of studying BAG1 protein localization.

**FIGURE 2:**
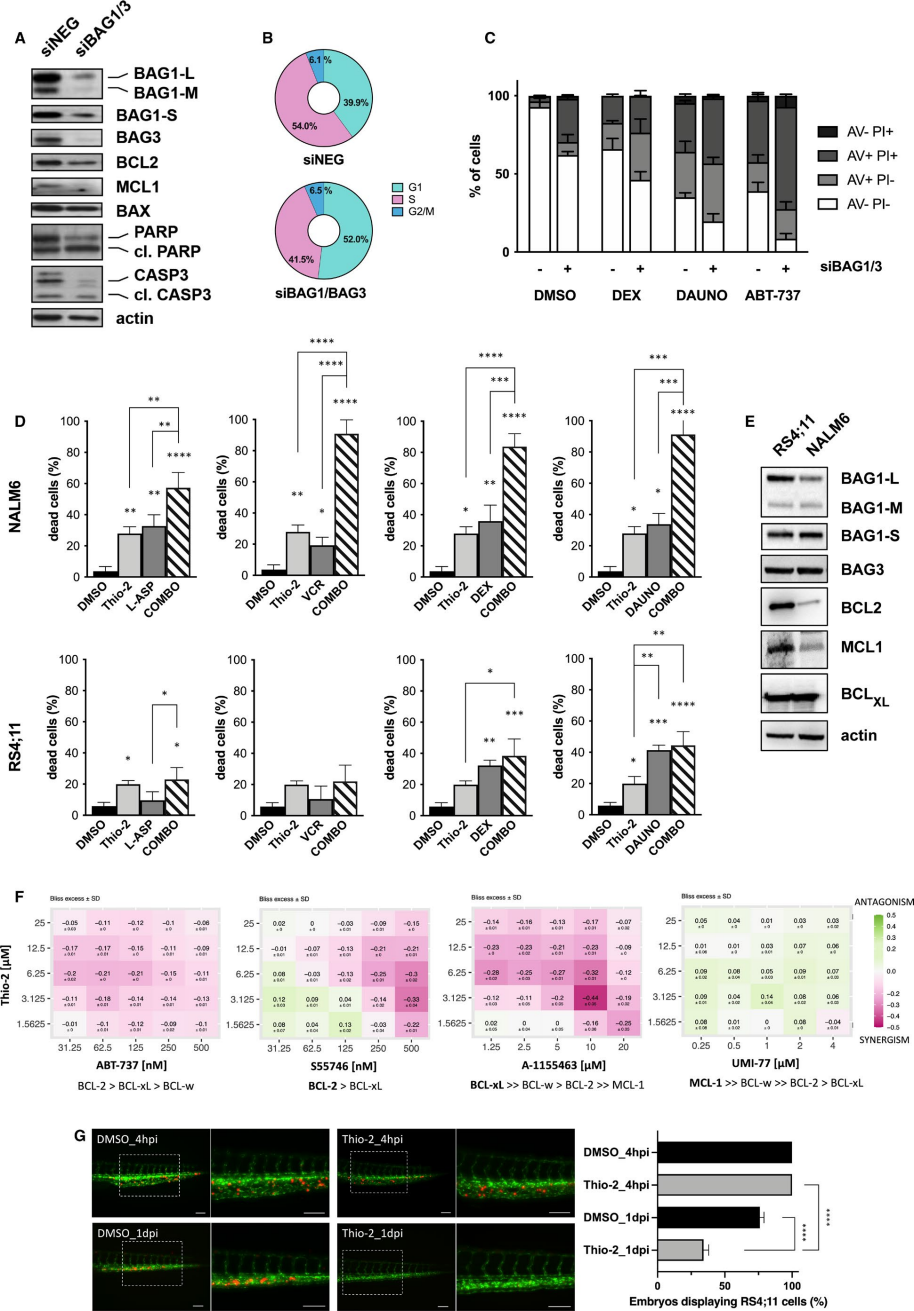
Targeting BAG1. The co‐silencing of BAG1/BAG3 (siBAG1/3) members in RS4;11 B‐ALL cell line activates the apoptotic pathway (A), induces G1‐phase cell cycle arrest (B) and sensitizes cells to conventional treatments as shown by annexin V‐propidium iodide (AV‐PI) cytofluorimetric analysis (C). Bars represent the mean ± SEM of three independent experiments. Two‐way ANOVA was performed to analyse statistical differences between siNEG vs. siBAG1/3 (DMSO: *p* < 0.001, DEX *p* = 0.002, DAUNO *p* = 0.01, ABT‐737 *p* < 0.001). (D) The BAG1 inhibitor Thio‐2 sensitizes B‐ALL cell lines to chemotherapeutic agents (L‐ASP 2UI, VCR 10 nM, DEX 0.1 μM, DAUNO 0.1 μM, Thio‐2 25 μM for NALM6) (L‐ASP 0.001UI, VCR 5 nM, DEX 5 nM, DAUNO 0.1 μM, Thio‐2 25 μM for RS4;11). Bars represent the mean ± SEM of three independent experiments. (E) Western blot analysis of BAG1, BAG3 and anti‐apoptotic protein level in NALM6 and RS4;11 cell lines. (F) Combination of Thio‐2 with ABT‐737 (pan‐BCL inhibitors) and agents selective for BCL2 (S55746) and BCL_XL_ (A‐1155463) exert a synergistic action in RS4;11 except for UMI‐77 (MCL1 inhibitor). Heat maps displaying the Bliss excess values computed for each point of the 5x5 combination treatment matrix designed. A positive Bliss excess value is indicative of compound antagonism (shades of green) while a negative Bliss excess value suggests compound synergism (shades of purple). (G) Confocal imaging of the trunk region (lateral view) of live Tg(Fli1:GFP) zebrafish embryos injected with 200 pre‐labelled RS4;11 cells (red signal). Blood vessels are stably marked with green (GFP). After 4 h from the xenotransplant, embryos were treated by adding Thio‐2 directly into the water at a final 25 μM concentration or vehicle (DMSO). After 24 h, Thio‐2–treated embryos showed a significantly reduced number of circulating RS4;11 cells compared to controls. ****, *p* < 0.001. Scale bar, 100 μm. Abbreviations: L‐ASP, L‐Asparaginase; VCR, Vincristine; DEX, Dexamethasone; DAUNO, Daunorubicine; hpi, hours post injection; dpi, days post injection

To gain insight into the mechanism by which BAG1 fulfils its cytoprotective role in B‐ALL, we adopted a small interfering RNA (siRNA) approach using the RS4;11 leukaemia cell line. Of note, following our previous experience, we used a combination of *BAG1*/*BAG3*‐specific siRNAs to prevent the functional rescue of BAG3 ^10^. The silencing of both BAG members in B‐ALL cells caused a decrease in the expression of anti‐apoptotic proteins BCL2 and MCL1, and only a slight decrease in BAX protein expression (Figure 2A). At the same time, *BAG1*/*BAG3* siRNA induced the cleavage of PARP and caspase 3 (Figure 2A), leading to a remarkable increase in the percentage of apoptotic (annexin V+) cells compared to the scrambled siRNA (siNEG) counterpart (35.8 ± 4.3 vs. 6.8 ± 2.9, respectively; *n* = 3; *p* < 0.001; Figure S2). In addition, *BAG1*/*BAG3* silencing induced the G1‐phase cell cycle blockade (Figure 2B). Together, these data suggest that, as previously reported for AML, even ALL cells benefit from the BAG1 over‐expression that assures their survival advantage over normal BM cell compartments.

BAG1 alone was able to define the susceptibility of B‐ALL cells to common chemotherapeutic drugs used in leukaemia therapy protocols. The knockdown of BAG1 was sufficient to sensitize RS4;11 cells to dexamethasone (DEX) and daunorubicin (DAUNO) by increasing significantly the proportion of apoptotic cells (Figure 2C and Figure S2). However, the strongest cytotoxic effect was observed upon treating BAG1‐depleted cells with the pan‐BCL inhibitor ABT‐737, pointing out the pivotal role of BAG1 in protecting BCL‐dependent regulation of the pro‐survival pathway in B‐ALL (Figure 2C). These observations highlight that BAG1 knockdown sensitizes B‐ALL cells to each of the drugs tested, strengthening the relevance of BAG1 protein in sustaining leukaemia cell survival. Moreover, those results underlined BAG1 targeting as a promising approach for the improvement of current B‐ALL treatment protocols. Nowadays, only a few compounds have been proposed as BAG1 inhibitors. Among them, the thioflavin S derivative Thio‐2 disrupts the interaction between BAG1 and HSP70 ^14^, ^15^, but also negatively modulates BAG1 and BCL2 protein levels in AML ^9^ and B‐ALL (Figure S3A). The treatment with Thio‐2 alone was sufficient to cause a significant cytotoxic effect in NALM6 and RS4;11 (Figure 2D), and it strongly enhanced the cytotoxic effect of L‐asparaginase (L‐ASP), vincristine (VCR), DEX and DAUNO in NALM6 (Figure 2D). Instead, RS4;11 cells were generally less responsive to the same drug combinations (Figure 2D). RS4;11 cells expressed higher levels of BAG1‐L isoform, BCL2 and MCL1 proteins compared to NALM6 (Figure 2E), suggesting an addiction to anti‐apoptotic mechanisms. Accordingly, RS4;11 cells were highly sensitive to pan‐BCL inhibitors (ABT‐737, navitoclax and venetoclax) and agents selective for BCL2 (S55746) and BCL_XL_ (A‐1155463), whereas NALM6 were resistant at the same dosages (Figure S3B; Table S2). More importantly, the combination of Thio‐2 with BCL inhibitors resulted in a synergistic effect, especially for S55746 and A‐1155463 (Figure 2F and Figure S3C). However, no synergistic effect has been observed for UMI‐77, a selective MCL1 inhibitor, confirming a strong BCL dependence for this B‐ALL cell line (Figure 2F).

To sustain additionally the efficacy of Thio‐2 against the growth of RS4;11 in vivo, we adopted a xenograft zebrafish model. Pre‐labelled RS4;11 cells were transplanted into the circulatory system of transgenic zebrafish embryos treated with 25µM Thio‐2 or vehicle. Notably, Thio‐2–treated embryos showed a significantly reduced number of circulating RS4;11 cells when compared to controls (Figure 2G), highlighting the cytotoxic effect of Thio‐2 *versus* leukaemia cells in vivo.

## 3 CONCLUSIONS

In the present study, we reported the correlation between the BAG1 protein levels and B‐ALL disease stage. We confirmed an over‐expression of BAG1‐L and BAG1‐M isoforms at ALL diagnosis and relapse with their concomitant decrease during the remission stage, where only the physiological BAG1‐S isoform remained detectable. Moreover, biological or chemical inhibition of endogenous BAG1 was sufficient to impair the survival of leukaemic cells while sensitizing them to chemotherapy. Yet, a large‐scale prospective cohort study will be mandatory to address whether BAG1‐L could be considered as a potential prognostic biomarker in B‐ALL or proposed for targeting in advanced leukaemia therapy protocols. Lastly, we place out a critical remark on the unmet needs for more effective BAG1 inhibitors that could implement therapeutic options for other malignancies with confirmed BAG1 deregulation.

## CONFLICT OF INTEREST

Part of the results of this paper was generated during a Ph.D. thesis (SA, Role of BCL2‐associated athanogene‐1 (BAG1) in acute myeloid leukaemia (AML): protein with hundred faces. Padova digital University archive). The authors declare that they have no other conflicts of interest.

## AUTHOR CONTRIBUTION

**Elena Mariotto:** Conceptualization (equal); Data curation (equal); Formal analysis (equal); Funding acquisition (equal); Methodology (equal); Validation (equal); Visualization (equal); Writing‐original draft (lead). **Diana Corallo:** Data curation (equal); Formal analysis (equal); Investigation (equal); Methodology (equal); Validation (equal); Visualization (equal); Writing‐original draft (equal). **Marcella Pantile:** Data curation (supporting); Methodology (supporting); Validation (supporting). **Emanuela Giarin:** Project administration (supporting); Resources (supporting); Validation (supporting). **Martina Pigazzi:** Supervision (supporting); Validation (supporting); Writing‐review & editing (supporting). **Giuseppe Basso:** Funding acquisition (supporting); Resources (supporting); Supervision (supporting). **Giampietro Viola:** Funding acquisition (equal); Resources (equal); Supervision (supporting); Writing‐review & editing (supporting). **Sanja Aveic:** Conceptualization (supporting); Data curation (supporting); Funding acquisition (supporting); Supervision (equal); Writing‐review & editing (supporting).

## Supporting information

Supplementary Material

## Data Availability

The data that support the findings of this study are available from the corresponding author upon reasonable request.
