# Supplementary material for: BAG1 down‐regulation increases chemo‐sensitivity of acute lymphoblastic leukaemia cells
Source: J Cell Mol Med. 2021 Aug 17;25(18):9060–5. doi: 10.1111/jcmm.16822 (PMC8435410; doi:10.1111/jcmm.16822)
Supplement: Supplementary file 1 — Supplementary Material [file JCMM-25-9060-s001.docx]

**MATERIALS AND METHODS**

**Cell lines**

RS4;11 and NALM6 human B-cell Acute lymphoblastic leukemia cell lines (all from American Type Culture Collection - ATCC) were cultured in RPMI 1640 supplemented with 10% inactivated FBS, 1 µg/mL of glutamine and 1 µg/mL of penicillin/streptomycin (GIBCO^®^ - Invitrogen; Carlsbad, CA) in HERAcell Incubator (Heraeus) at 37°C, 5% CO_2_ and saturated humidity (96% rH). B-ALL cell lines were cultured at 37°C, 5% CO2 for no longer than 15 passages. Mycoplasma testing was periodically performed using Venor GeM OneStep Mycoplasma Detection Kit (Minerva Biolabs, Berlin, Germania).

**B-cell Acute Lymphoblastic Leukemia (B-ALL) patients**

The expression of the BAG1 isoforms was analyzed in the bone marrow aspirates collected at diagnosis, remission, or relapse stage of pediatric B-ALL. Leukemia diagnosis was performed according to standard morphologic criteria based on immunohistochemical, immunophenotyping, and cytogenetic studies following the ALL-2000 treatment protocols (Conter *et al*., 2014). For this study, informed consent from parents in compliance with the Helsinki protocol was obtained.

**Transient transfection assay**

RS4;11 leukemia cells were co-transfected with exogenous small interfering RNAs (siRNA) specifically recognizing BAG1 (350 pmol) and BAG3 (200 pmol - Santa Cruz Biotechnology, Santa Cruz, CA) using the Amaxa Nucleofactor systems (Lonza; Cologne, Germany), with L-017 program and according to manufacturer’s protocol. An equivalent amount of non-silencing scramble siRNA (sc-siRNA; Santa Cruz Biotechnology, Santa Cruz, CA) was used as a control.

**Reagents, apoptosis assay and cell cycle analysis**

Cell death activation was achieved by administration of cytotoxic compounds at the indicated concentrations and time points with the following compounds: vincristine sulfate (Tocris Bioscience, Bristol, UK), daunorubicin (Pfizer, New York, NY), dexamethasone (Sigma-Aldrich, Milan, Italy), L-Asparaginase (Santa Cruz Biotechnology), ABT-737 (Selleck Chemicals, Munich, Germany), Thio-2 (AKos GmbH, Germany). Measurement of cell death was done using fluorescein-labeled Annexin-V and Propidium Iodide with Annexin-V FLUOS Staining kit (Roche Diagnostics, Basel, Switzerland) and according to manufacturer’s instruction. Samples were analyzed on a Cytomics FC500 flow cytometer (Beckman Coulter, Brea, CA). The BAG1 inhibitor Thio-2 was used at 25 μM concentration. NALM6 cells (L-ASP 2UI, VCR 10nM, DEX 0.1μM, DAUNO 0.1μM, Thio-2 25μM) RS4;11 (L-ASP 0.001UI, VCR 5nM, DEX 5nM, DAUNO 0.1μM, Thio-2 25μM). For statistical purposes, 10000 events were acquired in the ungated population in three independent replicates.

**Cell cycle distribution analysis**

For flow cytometric analysis of DNA content, 5×10^5^ cells transiently transfected with siRNA for BAG1 and BAG3 or with sc-siRNA were collected, centrifuged, and fixed with ice-cold ethanol (70%). The cells were then treated with a buffer containing RNAse A (Qiagen, Hilden, Germany) and 0.1% Triton X-100 (Sigma-Aldrich), and then stained with propidium iodide (PI) (Sigma-Aldrich). Samples were analyzed on a Cytomics FC500 flow cytometer (Beckman Coulter, Brea, CA). For statistical purposes, 25000 events were acquired in the singlets population (AUX vs. FL3-H) in three independent replicates. DNA histograms were analyzed using MultiCycle for Windows (Phoenix Flow Systems; USA).

**Preparation of total protein extracts and sub-cellular protein fractions**

Total protein lysates were isolated using commercially available lysis buffer (Biosource International; Camarillo, CA), supplemented with 0.5 mM PhenylMethaneSulfonyl Fluoride (PMSF, Sigma-Aldrich), 1X mammalian protease, and 1X phosphatase inhibitor mix (Sigma-Aldrich). For the extraction of cytosol and nuclear protein fractions, a combination of two buffers, low salt (10 mM HEPES pH = 7.9; 1.5 mM MgCl2; 10 mM KCL; and 0.5 mM DTT), and high salt (20 mM HEPES pH =7.9; 25% v/v glycerol; 0.42 M NaCl; 1.5 mM MgCl2; 0.2 mM EDTA; and 0.5 mM DTT) was used (Aveic S. *et al.,* 2011). Protein quantification was done using BCA Protein Assay Kit (Pierce; Rockford, IL) according to the manufacturer's instructions.

**Western blotting**

Aliquots of the total, nuclear, or cytosol lysates (20 μg) were subjected to sodium dodecyl sulfate-polyacrylamide gel electrophoresis (SDS-PAGE). Immunoblots were hybridized with the following primary antibodies: BAG1 (recognizing all three BAG1 isoforms), BAG3 (SC Biotechnology), CASPASE-3, PARP, BCL2, MCL1, BAX, BCL_XL_ (Cell Signaling; Danvers, MA). Blots were then stripped and re-probed with β-ACTIN (Sigma-Aldrich; Italy) or HDAC1 (SC Biotechnology) antibodies used as the protein loading controls. As secondary antibody horseradish peroxidase (HRP)–conjugated anti-rabbit or anti-mouse IgG (Upstate Biotechnology; Lake Placid, NY) were used. Enhanced chemiluminescence (ECL) Western blotting detection reagents and films (GE Healthcare; UK) were used for protein bands acquisition and, densitometry was done using ImageJ 1.37v software (National Institutes of Health; Bethesda; MD).

**Dose-response curves and drug combination experiments**

The potential synergistic/antagonistic behavior of the BAG1 inhibitor Thio-2 when combined with pan-BCL inhibitors (ABT-737, Navitoclax, and Venetoclax) or agents selective for BCL2 (S55746), BCL_XL_ (A-1155463), and MCL1 (UMI-77) (Selleck Chemicals, Houston, TX) was evaluated in RS4;11 cells according to the Bliss excess method. Briefly, 10000 cells/well were seeded in 384-well plates and, after 24 h, they were pre-treated with Thio-2 at the indicated doses and then treated with BH3 mimetics the day after for an additional 48 h resulting in a 5x5 drug combination matrix. After 72 h of co-treatment, cells were assayed by a resazurin-based cell viability test. To ensure treatment reproducibility, all procedures, including cell seeding, drug dilution, cell treatment, and application of resazurin solution were carried out through a 96-channel robotic liquid handler (Microlab STAR 96-CORE, Hamilton, Bonaduz, GR, Switzerland). The Bliss excess was then computed for all technical replicates per dose per drug combination. Data were processed using R 3.6.3 and RStudio Version 1.3.1056 according to Flobak *et al*. 2019.

**Zebrafish xenotransplantation model**

The Tg(fli1:EGFP) zebrafish embryos (Stoletov *et al*., 2007) were raised, staged, and maintained as already described (Kimmel et al., 1995). Dechorionized, two days post fertilization (dpf) zebrafish embryos were anesthetized with 0.003% tricaine (Sigma-Aldrich) and positioned on a 10 cm Petri dish coated with 3% agarose. Non-fluorescent RS4;11 cells were labeled with the Vybrant® DiI Cell-Labeling Solution (Invitrogen) according to the manufacturer’s instructions. Cells were then implanted using borosilicate glass capillary needles (OD/ID: 1.0/0.75 mm, WPI), a Pneumatic Picopump, and a micro-manipulator (WPI). Approximately 200 cells were injected within the duct of Cuvier of each anesthetized embryo. After the implantation, zebrafish embryos were maintained at 33 °C. The animals showing less than 100 cells after 2 h post-injection were discarded from the analysis. At least 50 embryos per group were analyzed from three independent experiments. Embryos were live photographed using a BM6000 (Leica) microscope equipped with a PerkinElmer UltraVIEW VoX Confocal System.

The Fondazione Istituto di Ricerca Pediatrica Città della Speranza (IRPCDS) is authorized to use rodents and zebrafish for biomedical research by the Ministry of Health with Ministerial Decree No. 21/2019-UT dated 4 July 2019 that replaces the previous Ministerial Decree No. 09/2014-UT dated 30 December 2014 according to D.lgs 26/2014, concerning the protection of animals used for scientific purposes.

**Statistical analysis**

Graphs and statistical analyses were performed using GraphPad Prism software (GraphPad, La Jolla, CA). All data in graphs represented the mean of at least three independent experiments ± standard error of the mean (SEM). Statistical significance was determined using Student t-test or ANOVA (one- or two-way) depending on the type of data. For multiple test comparisons, Bonferroni or Newman-Keuls corrections were applied. Asterisks indicate a significant difference between the treated and the control group unless otherwise specified. **p<0.05, **p<0.01, ***p<0.001, ****p<0.0001.*

**Supplementary Figure S1**

**
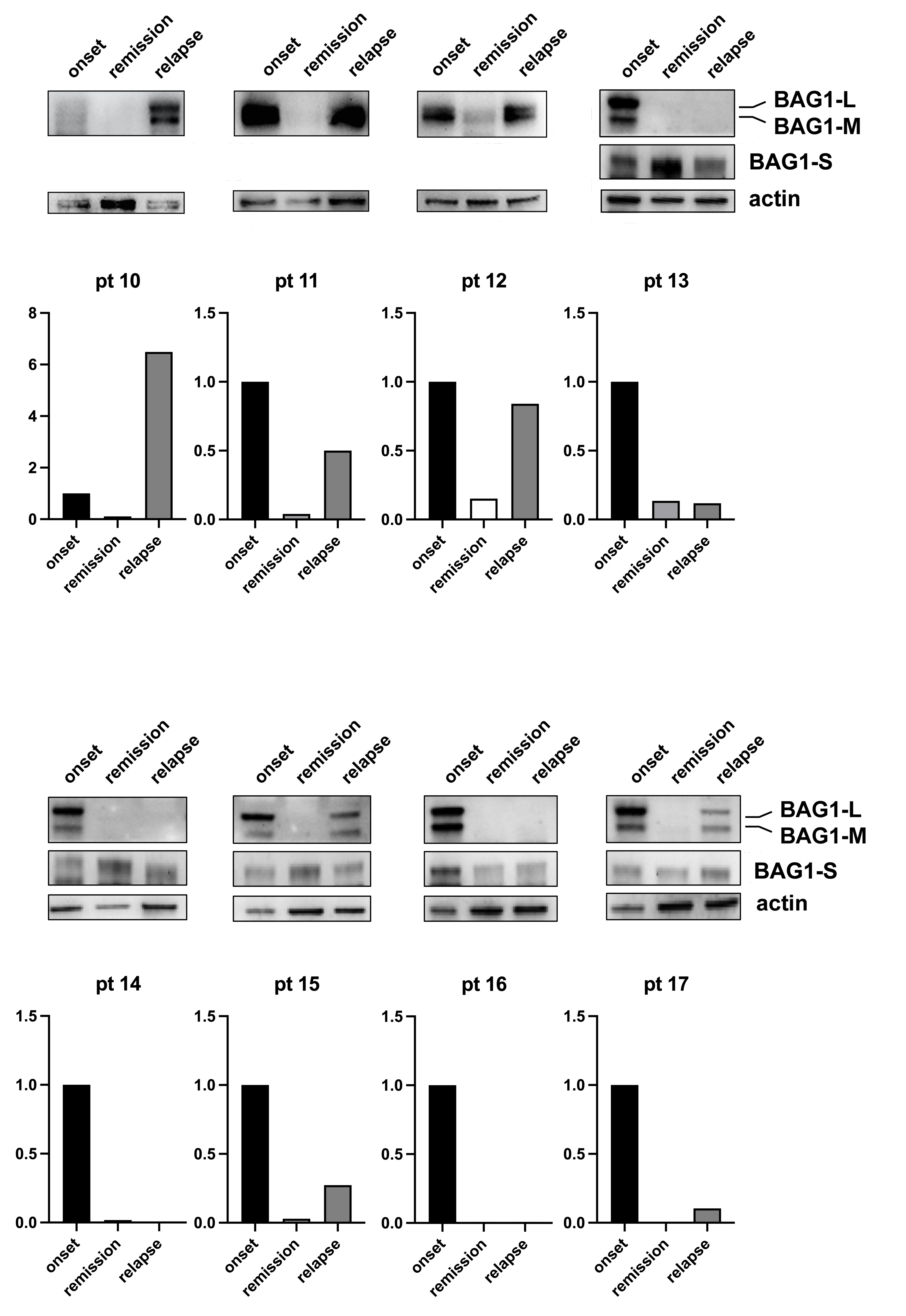
**

**Figure S1. BAG1-S inclusion to the main Figure 1.** Protein expression in different stages of B-ALL is shown as an implementation to the main Figure 1. Representative Western Blot analysis are showing not only BAG1-M, BAG1-L, but where possible also BAG1-S, isoforms in paired B-ALL specimens collected at disease onset, remission and relapse. The relative densitometry analysis has been performed by normalizing the whole BAG1-L expression over the loading control (actin) and data are expressed as fold-increase respect to the onset phase.

**Supplementary Figure S2**


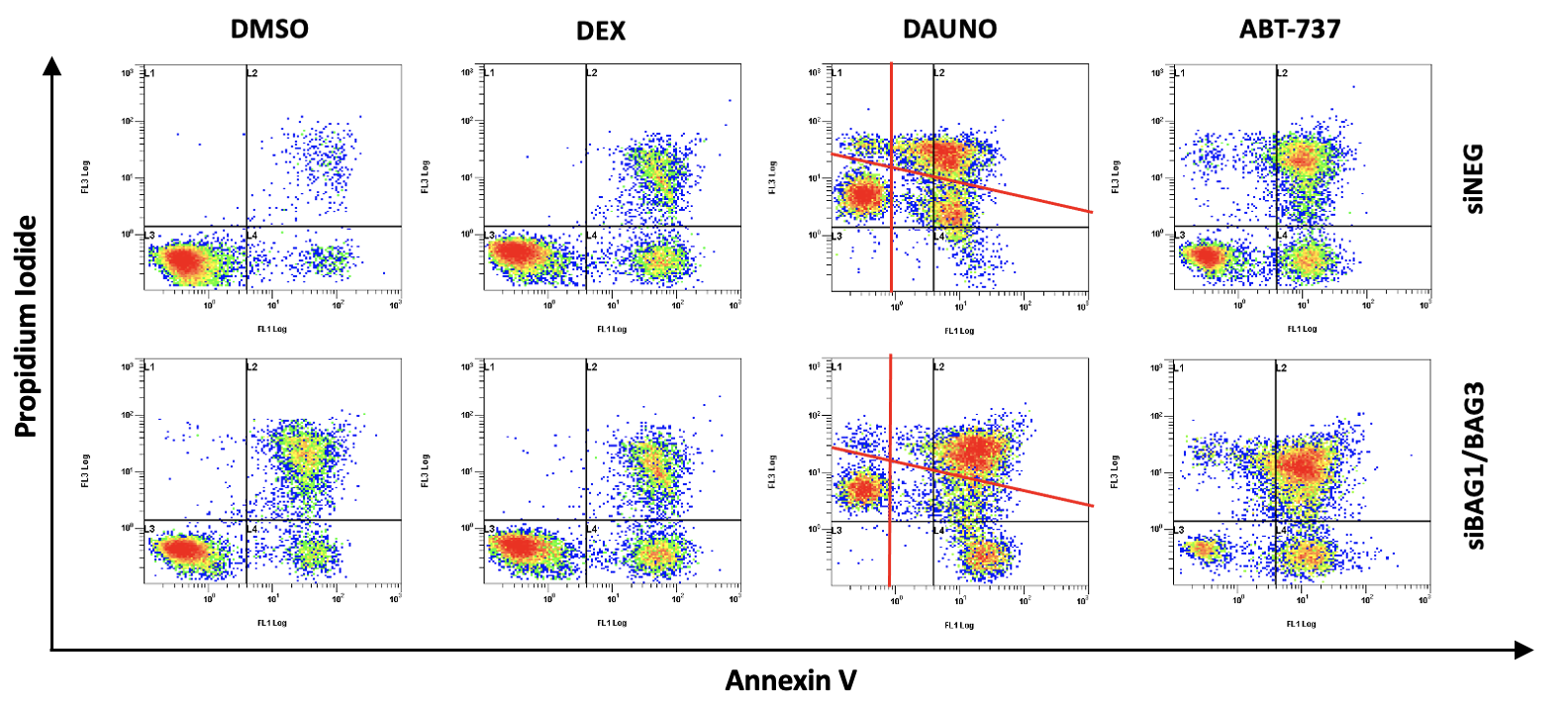


**Figure S2. BAG1/BAG3 co-silencing sensitizes RS4;11 cells to common therapeutics**

Representative bi-parametric analysis of AnnexinV/Propidium Iodide (AV/PI) staining performed in RS4;11 cells after 24 h of silencing with scramble (siNEG) or siBAG1/BAG3 and subsequently treated with the indicated compounds for additional 48 h. Of note, the gating strategy has been changed for DAUNO treatment (red axes) taking into account the red fluorescence (FL3) of DAUNO itself, which however remained distinguishable from PI fluorescence. Abbreviations: DEX, Dexamethasone; DAUNO, Daunorubicine.

**Supplementary Figure S3**


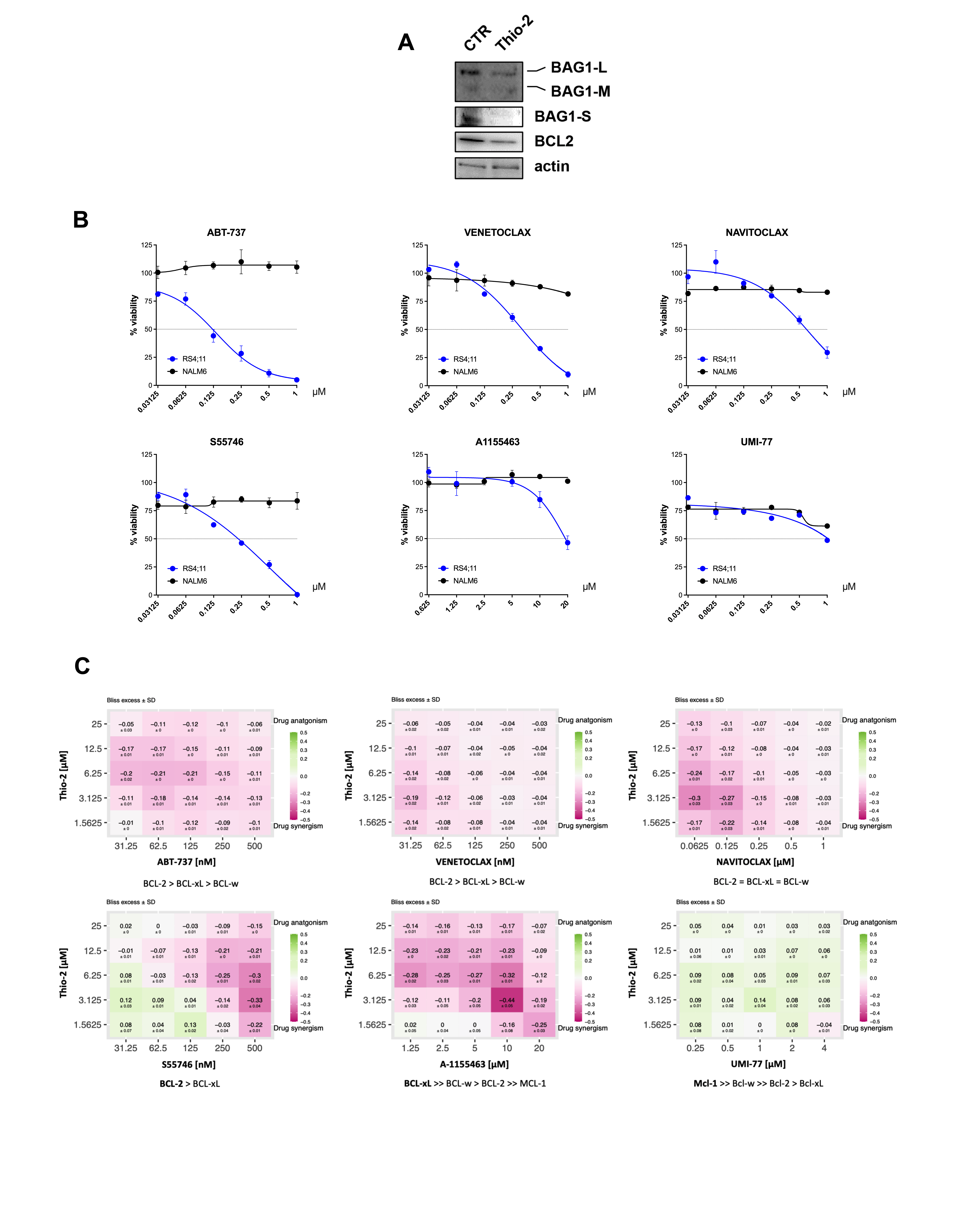


**Figure S3. Evaluation of Thio-2 treatment in B-ALL cell lines**

**(A)** Thio-2 treatment reduces BAG1 isoforms and BCL2 levels in RS4;11 cell line. **(B)** Relative proliferation/viability of RS4;11 and NALM6 after exposure with scalar doses of indicated BH3 mimetics for 48 h as measured by a resazurin-based assay. (**C)** Heatmaps displaying the Bliss excess values calculated in each point of the 5x5 combination treatment matrix. A positive Bliss excess value is indicative of compound antagonism (shades of green) while a negative Bliss excess suggests compound synergism (shades of purple).

**Supplementary Table S1. Characteristics of B-ALL patients used in this study.**

| Patient # | Gender | Age at diagnosis (years) | % of blasts in BM | Cytogenetic | DNA index |
| --- | --- | --- | --- | --- | --- |
| 1 | F | 5 | 86 | N/A | N/A |
| 2 | F | 8 | 53 | N/A | 1.2 |
| 3 | M | 3 | UD | N/A | 1 |
| 4 | M | 2 | UD | 12;21 | 1 |
| 5 | F | 15 | 92 | N/A | 1.93 |
| 6 | F | 2 | 94 | NEG | 1 |
| 7 | M | 2 | 66 | 11q23RR | 1 |
| 8 | M | 3 | 95 | NEG | UD |
| 9 | F | N/A | N/A | N/A | N/A |
| 10 | M | 3 | 77 (87)* | 12;21 | 1 |
| 11 | M | 11 | 86 (92)* | 9;22 | 1 |
| 12 | M | 3 | 90 (92)* | 12;21 | 1.6 |
| 13 | M | 5 | 70 (N/A)* | NEG | 1 |
| 14 | M | 4 | 94 (N/A)* | NEG | 1 |
| 15 | M | 2 | 92 (90)* | NEG | 1 |
| 16 | M | 2 | 95 (73)* | 11;19 | 1 |
| 17 | M | 2 | 83 (85)* | NEG | 1 |

*First value refers to the blast % at disease onset; Value between the brackets indicates % of blasts at relapse. N/A – not available; UD – undetermined during analysis; RR- rearrangement.

**Supplementary Table S2. Characteristics of BH3 mimetics used in this study.**

| Inhibitors | Target Specificity (K_i_) | | | | GI_50_ |
| --- | --- | --- | --- | --- | --- |
|  | **BCL2** | **BCL_XL_** | **BCLw** | **MCL1** | **RS4;11 (µM)** |
| ABT-737 | 30.3 nM | 78.7 nM | 197.8 nM | N/A | 0.13 ± 0.03 |
| NAVITOCLAX | ≤1 nM | ≤ 0.5 nM | ≤1 nM | N/A | 0.60 ± 0.12 |
| S55746 | 1.3 nM | 520 nM | N/A | N/A | 0.23 ± 0.04 |
| A-1155463 | 80 nM | < 0.01 nM | 19 nM | > 440 nM | 18.02 ± 0.87 |
| UMI-77 | 23.83 µM | 32.99 µM | 8.19 µM | 0.49 µM | 1.01 ± 0.11 |

- Target Specificity (K_i_) declared from vendor for the indicated compounds, and relative growth inhibitory concentration (GI_50_) calculated for RS4;11 cells after 48 h of treatment (see Figure S2B). N/A - not available.
